# Soft tissue surgical robot for minimally invasive surgery: a review

**DOI:** 10.1007/s13534-023-00326-3

**Published:** 2023-10-13

**Authors:** Minhyo Kim, Youqiang Zhang, Sangrok Jin

**Affiliations:** School of Mechanical Engineering, Pusan National University, 2, Busandaehak-ro 63beon-gil, Geumjeong-gu, Busan, 46241 Republic of Korea

**Keywords:** Surgical robot, Robot-assisted surgery, Laparoscopic surgery, Endoluminal surgery, Single-port surgery

## Abstract

**Purpose:**

The current state of soft tissue surgery robots is surveyed, and the key technologies underlying their success are analyzed. State-of-the-art technologies are introduced, and future directions are discussed.

**Methods:**

Relevant literature is explored, analyzed, and summarized.

**Results:**

Soft tissue surgical robots had rapidly spread in the field of laparoscopic surgery based on the multi-degree-of-freedom movement of intra-abdominal surgical tools and stereoscopic imaging that are not possible in conventional surgery. The three key technologies that have made surgical robots successful are wire-driven mechanisms for multi-degree-of-freedom movement, master devices for intuitive remote control, and stereoscopic imaging technology. Recently, human-robot interaction technologies have been applied to develop user interfaces such as vision assistance and haptic feedback, and research on autonomous surgery has begun.

**Conclusion:**

Robotic surgery not only replaces conventional laparoscopic surgery but also allows for complex surgeries that are not possible with laparoscopic surgery. On the other hand, it is also criticized for its high cost and lack of clinical superiority or patient benefit compared to conventional laparoscopic surgery. As various robots compete in the market, the cost of surgical robots is expected to decrease. Surgical robots are expected to continue to evolve in the future due to the need to reduce the workload of medical staff and improve the level of care demanded by patients.

## Introduction

Minimally invasive surgery is an irresistible megatrend of recent advances in surgical techniques. Since the end of the 20th century, open abdominal surgery has been rapidly replaced by laparoscopic surgery [1]. Laparoscopic surgery is performed without opening the abdomen, through a passageway created by several small incisions, usually less than 15 mm in diameter, through which an endoscopic camera and specialized elongated surgical instruments are inserted. The smaller wound created by the surgery results in less bleeding, less risk of infection, and a shorter recovery period. Patients experience less pain and less scarring [2]. In addition to these clinical advantages, high patient satisfaction has led to its widespread application in gynecologic surgery such as hysterectomy, general surgery such as cholecystectomy and colorectal cancer surgery, and urologic surgery [3, 4]. More recently, single-port laparoscopic surgery, in which surgery is performed through a single passage, has also been performed. In the field of gynecology, laparoscopic surgery is frequently performed due to its distinct advantages from a cosmetic point of view.

Laparoscopic surgery is performed by viewing the image transmitted through an endoscope camera rather than directly observing the surgical site. It is difficult to see a wide range of searches due to observing only a narrow space, and flat-screen images lack three-dimensional information such as distance. Because the surgery is performed with a long, straight surgical instrument through a trocar, with only four degrees-of-freedom of motion: forward, backward, and rotation, movement is limited compared to open surgery, where hands are used to operate directly inside the abdominal cavity. Tactile sensation is also desensitized due to the large distance between the tip of the surgical tool and the operating hand. The above disadvantages greatly increase the difficulty of surgery, making the outcome highly dependent on the skill of the surgeon and making it impossible to perform complex surgeries that were previously performed in open surgery [5].

Launched after receiving FDA (Food and Drug Administration) clearance in 2000, Intuitive Surgical Inc. (U.S.)’s da Vinci Si is a surgical robot that combines the benefits of laparoscopic surgery while addressing its drawbacks [6]. It uses a binocular endoscopic camera and an eyepiece display on the console to create a stereoscopic view, providing the same sense of distance as open surgery. The surgical tools are equipped with a wrist-type mechanism driven by a wire, allowing various movements like a surgeon’s hand in open surgery. In addition, the surgeon can intuitively operate the robotic arm through a master device on the control console, enabling complex surgeries. Through the control technology, the tremor of the operator’s hand is filtered, and scalable motion is available, enabling precise and safe surgery that is difficult to achieve in conventional surgery [7]. Robotic surgery is not only replacing conventional laparoscopic surgery but also enabling complex surgeries that are not possible in laparoscopic surgery. Recently, rather than adapting robots to existing surgeries, on the contrary, surgical techniques tailored to robots have been developed and published [8]. In line with the development direction of minimally invasive surgery, the da Vinci SP, which can perform single-hole surgery, has been developed and entered the market [9].

As more cases of robotic surgery are accumulated and analyzed, more neutral research on the efficacy of surgical robots is emerging [10]. The disadvantages of robotic surgery include high costs and increased operative time. While there are some procedures, such as prostatectomy, where robotic surgery has clear advantages [11], there are also a growing number of reports of no clinical difference or patient benefit compared to conventional laparoscopic surgery for relatively simple procedures such as cholecystectomy and right colectomy [12]. Currently, the laparoscopic surgical robot market is dominated by Intuitive Surgical Inc.‘s Da Vinci System. However, with the recent expiration of key patents, various companies are developing and launching laparoscopic surgical robots [13]. As more robots compete in the market, the cost of surgical robots is expected to decrease. Robotic technology is constantly evolving to provide safer and higher-quality surgeries. In recent years, rather than improving the mechanical performance of robots, research and development have been focused on user interfaces to provide surgeons with rich and accurate information and easier operation. In addition, research on autonomous surgery using robots continues.

This review paper introduces various surgical robots and describes their core technologies, focusing on laparoscopic surgical robots that handle flexible tissues. The current state of the art is reviewed, and the future direction of development is discussed.

## Overview of surgical robot for soft tissue surgery

The success of the da Vinci system has led to the development and market introduction of a variety of subsequent surgical robots, from multi-port surgical robots, which operate through multiple incisions in the body, to single-port surgical robots, which operate through a single orifice, following a philosophy about minimally invasive surgery. Furthermore, flexible surgical robots have emerged, which have a flexible structure and perform surgeries or procedures through natural human orifices. In this paper, we have divided the robot’s structure into rigid and flexible tubes. A tube in a surgical robot is defined as a structure that is inserted into the body via a trocar. A rigid tube is a structure that is inserted into the body through a rigid straight tube and a flexible tube is a structure that is inserted into the body through a soft structure. While there are many different robots out there, we prioritized listing the ones that are licensed to the best of our knowledge. Table 1 summarizes information about representative surgical robots. We searched for information using Scholar Google based on robot names, focusing on licensed robots. All information was retrieved by June 15th, 2023.

**Table 1:** Summary of soft tissue surgical robots

Product	Release	Company/Country	Specialization	Configuration	Clearance
da Vinci Xi	2014	Intuitive Surgical Inc. U.S.	Urology, General Surgery, Gynecology, Otorhinolaryngology, Thyroid Diseases [6]	Rigid type with four arms on one cart for multi-port surgery	FDA, CE [6, 60]
da Vinci SP	2018	Intuitive Surgical Inc. U.S.	Urology, TORS procedures [27, 30]	Rigid type with four wire-driven snake-type arms on one overtube for single-port surgery	FDA [30]
Versius	2019	CMR Surgical Ltd. U.K.	Gynecology, Colorectal Surgery, Thoracic Surgery, General Surgery, Urology [6, 15]	Rigid type with one arm on one cart for multi-port surgery with multiple robots in a modular configuration	CE [6]
Hugo™ RAS	2022	Medtronic U.S.	Urology, Gynecology [20, 21]	Rigid type with one arm on one cart for multi-port surgery with multiple robots in a modular configuration	CE [24]
Senhance®	2016	Asensus Surgical Inc. U.S.	General Surgery, Gynecology [6]	Rigid type with one arm on one cart for multi-port surgery with multiple robots in a modular configuration	FDA, CE [6, 58]
Revo-i	2017	Meere Company Inc. South Korea	Urology, Gynecology, ENT, Colorectal and Anal Surgery [23]	Rigid type with four arms on one cart for multi-port surgery	MFDS [13]
Avatera	2019	avateramedical® GmbH Germany	Urology, Gynecology [24]	Rigid type with four arms on one cart for multi-port surgery	CE [24]
Hinotori	2020	Medicaroid Inc. Japan	Urology [25]	Rigid type with four arms on one cart for multi-port surgery	MHLW, HAS [59]
Toumai	2022	Shanghai MicroPort MedBot Co., Ltd. China	Urology [26]	Rigid type with four arms on one cart for multi-port surgery	NMPA [26]
Flex Robotic System	2017	Medrobotics Corp. U.S.	Natural Orifice surgery [33]	One flexible robotic endoscope and two flexible manual surgical instruments for ELS [36]	FDA, CE [6, 33]
Ion Endoluminal System	2019	Intuitive Surgical Inc. U.S.	Biopsy in the Peripheral Lung [36]	Flexible type for endoluminal biopsies	FDA [36]
MONARCH Platform	2018	Ethicon Inc. U.S.	Biopsy in the Peripheral Lung, Urology [36]	Flexible type for endoluminal biopsies	FDA [36]

**Clearance**: FDA: U.S. Food and Drug Administration; CE: Conformité Européene; MFDS: The Korean Ministry of Food and Drug Safety; MHLW: Japanese Ministry of Health, Labour and Welfare; HSA: Singapore Health Sciences Authority; NMPA: China National Medical Products Administration

### Surgical robot with rigid tube for multi-port surgery

**Fig. 1:**
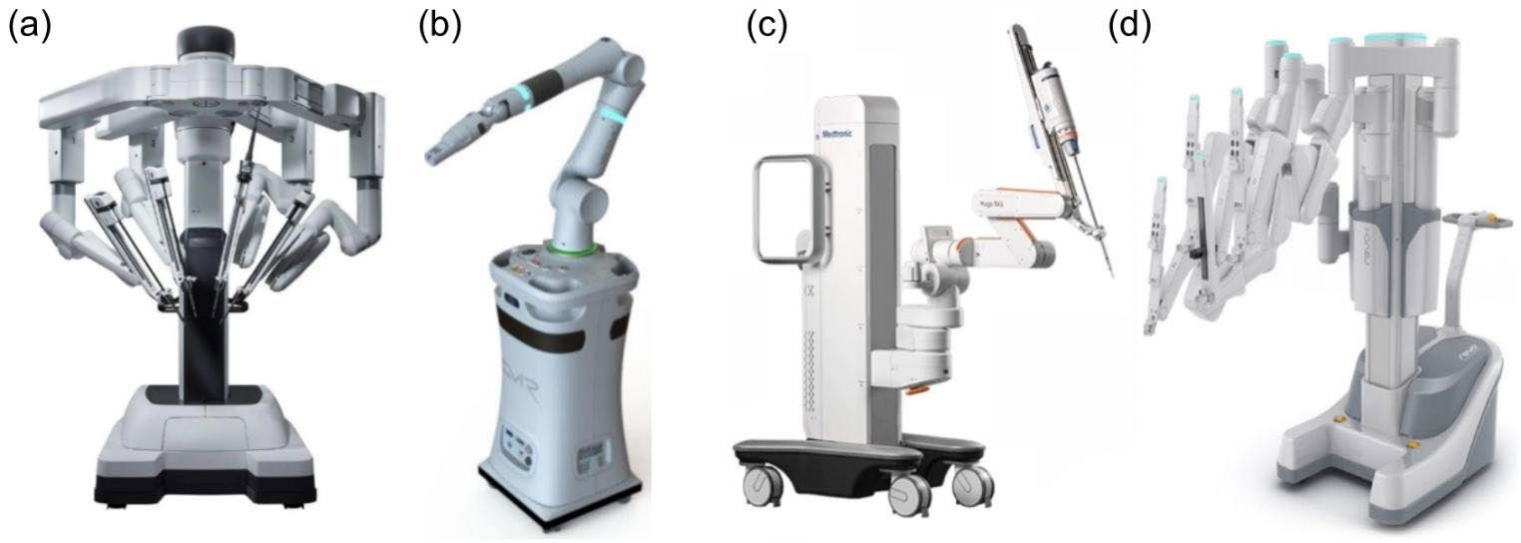
Rigid-type surgical robot for multi-port surgery; **(a)** da Vinci Xi [28], **(b)** Versius [16], **(c)** Hugo™ RAS [21], **(d)** Revo-i [60]

Intuitive Surgical Inc. has released a series of robots, starting with the initial version, the da Vinci Si, followed by the da Vinci Xi (Fig. 1(a)), the most advanced robot, and the da Vinci X, an entry-level robot with reduced features and a lower price. The da Vinci Xi moves the robotic arm overhead to allow access to more areas of the body. Like the older Si model, da Vinci X limits access to the surgical object because the robotic arm is mounted on a side cart. Today, the da Vinci system has sold more than 6,000 units worldwide and has recorded more than 8.5 million surgeries [14]. All examples of laparoscopic surgery, prostatectomy, cholecystectomy, and so on mentioned in the introduction, were all performed by the da Vinci system. Its overwhelming advantage over other surgical robots is that it has developed an extensive lineup of robotic surgical tools, including various types of graspers, needle drivers, and scissors, as well as energy devices, suction, and irrigation. It also holds clearances applicable to a variety of surgical specialties, including urology, gynecology, general surgery, cardiac surgery, and otolaryngology. However, it is being challenged by various surgical robotics companies as key patents expire.

The Versius (Fig. 1 (b)) is a surgical robot developed by CMR Surgical Ltd. (U.K.) that features a portable and modular design. While the da Vinci has all four robotic arms mounted on one base cart, the Versius has one robotic arm mounted on one cart. Multiple robots can be positioned around the operating table to perform the surgery [15, 16], making efficient use of space in small operating rooms. They focused on improving the user interface compared to the da Vinci system [17]. With an open console, it is easy to communicate with the surrounding medical staff while controlling the robot, and it also has the performance of a collaborative robot that allows medical staff to intervene and interact with the robot during surgery. Currently, it is accumulating surgical cases in Europe after obtaining CE (Conformite Europeenne) [18].

The Hugo™ RAS (Fig. 1 (c)) is a robot released by Medtronic (U.S.), a well-known global medical device company, and has a portable and modular structure similar to the Versius. The main feature is the haptic feedback function that allows the operator to feel the rebound force of soft tissue [19]. It is accumulating surgical cases in gynecologic and urologic surgery [20, 21].

The Senhance® is a robot developed by Asensus Surgical Inc. (U.S.) that features an eye-tracking camera. It can recognize the user’s eye movements and manipulate the endoscopic camera accordingly, improving control, reducing distractions, and optimizing visualization during surgery. It is available in the U.S. and Europe and has been applied to prostate surgery [22].

The Revo-I (Fig. 1 (d)) is a surgical robot developed by Meere Company Inc. (South Korea) and was the second surgical robot in the world at the time, after da Vinci. It has a similar structure to da Vinci Si and can perform surgical operations such as incision, cutting, electrocautery, suturing, insertion, and fixation It was applied to prostate surgery [23] and more, achieving a record of more than 100 domestic surgeries in 2021.

In addition, surgical robots similar to the da Vinci system, such as Avatera (avateramedical® GmbH) [24] in Germany, Hinotori (Medicaroid Inc.) [25] in Japan, and Toumai (Shanghai MicroPort MedBot Co., Ltd.) [26] in China, have been developed and released in each country.

### Surgical robot with rigid tube for single-port surgery

**Fig. 2:**
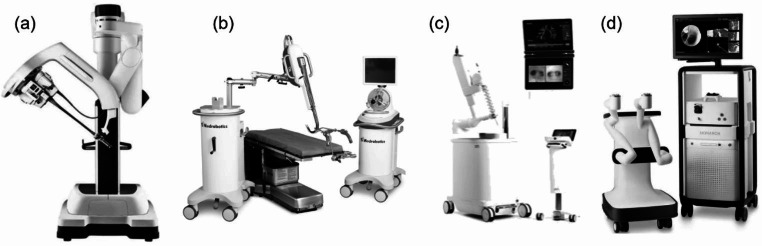
Surgical robot for single-port surgery and flexible robot for endoluminal surgery; **(a)** da Vinci SP [28], **(b)** Flex Robotic System [6], **(c)** Ion Endoluminal System [34], **(d)** MONARCH Platform [35]

Recently, many laparoscopic surgeries have been performed with a single port, mainly in gynecology, by mounting a multi-port cover on a single wound protector. The da Vinci system also enables single-site surgery by crossing the robotic arms [27, 28]. The da Vinci SP (Fig. 2 (a)), released in 2017, goes even further, with four wire-driven robotic arms inserted through a single overtube, using one as a camera and three as surgical tools to perform single-port surgery. In addition to conventional abdominal surgery [29], surgery through natural orifices such as transoral or transanal surgery is also possible [30, 31].

### Surgical robot with flexible tube for endoluminal surgery

The development of flexible robots has opened up new possibilities in the field of surgical robotics. Flexible surgical robots can enter through natural orifices such as the bronchi, nasal passages, and urethra and traverse complex pathways to access surgical and procedural sites. Combined with image guidance and navigation technologies, they have the potential to advance high-level endoscopic procedure. It is hoped that the robot’s payload can be improved and expanded to the gastrointestinal field in the future [32]. Since the late 2000s, NOTES (Natural Orifice Transluminal Endoscopic Surgery) research utilizing flexible surgical robots has been actively studied, and recently, the concept has been slightly changed to ELS (Endoluminal Surgery).

The Flex Robotic System (Fig. 2 (b)) was developed by Medrobotics Corp. (U.S.) and received FDA clearance for ENT (ear, nose, and throat) in 2015. A flexible endoscope driven by a robot is remotely controlled by the surgeon, and flexible surgical instruments are inserted along the side guides, allowing the operator to perform the surgery manually. It shows superior accessibility compared to conventional surgical robots in transoral surgery [33].

Developed by Intuitive Surgical Inc. following da Vinci system, the Ion Endoluminal System (Fig. 2 (c)) is a flexible endoscopic robot for bronchoscopy-assisted and peripheral lung biopsy. It received FDA clearance in 2019. It utilizes shape detection technology for easy localization. It can accurately access and biopsy lesions and reduce examination time [34].

The MONARCH Platform (Fig. 2 (d)) is a flexible robot for the diagnosis and biopsy of lung lesions, similar to the Ion Endoluminal System. It was developed by Auris Health Inc. (U.S.) and received FDA clearance in 2018. In 2019, Johnson & Johnson, a global medical device company, acquired Auris Health Inc. and the robot is now handled by its subsidiary Ethicon Inc. In 2022, it also received FDA approval for urology, making it the first flexible surgical robot with multispecialty capabilities. A study evaluating its performance compared to the Ion Endoluminal System was reported [35, 36].

## Key technologies

Three technologies have led to significant advances over conventional laparoscopic surgery and have contributed to the widespread adoption of robotic-assisted surgery. These are wire-driven joints that enable multi-degree-of-freedom movement within the abdominal cavity, a remote-controlled master device that allows intuitive manipulation of the surgical tools as if they were done by the surgeon’s hands, and finally, vision technology that provides crisp three-dimensional images.

### Wire-driven manipulator

**Fig. 3:**
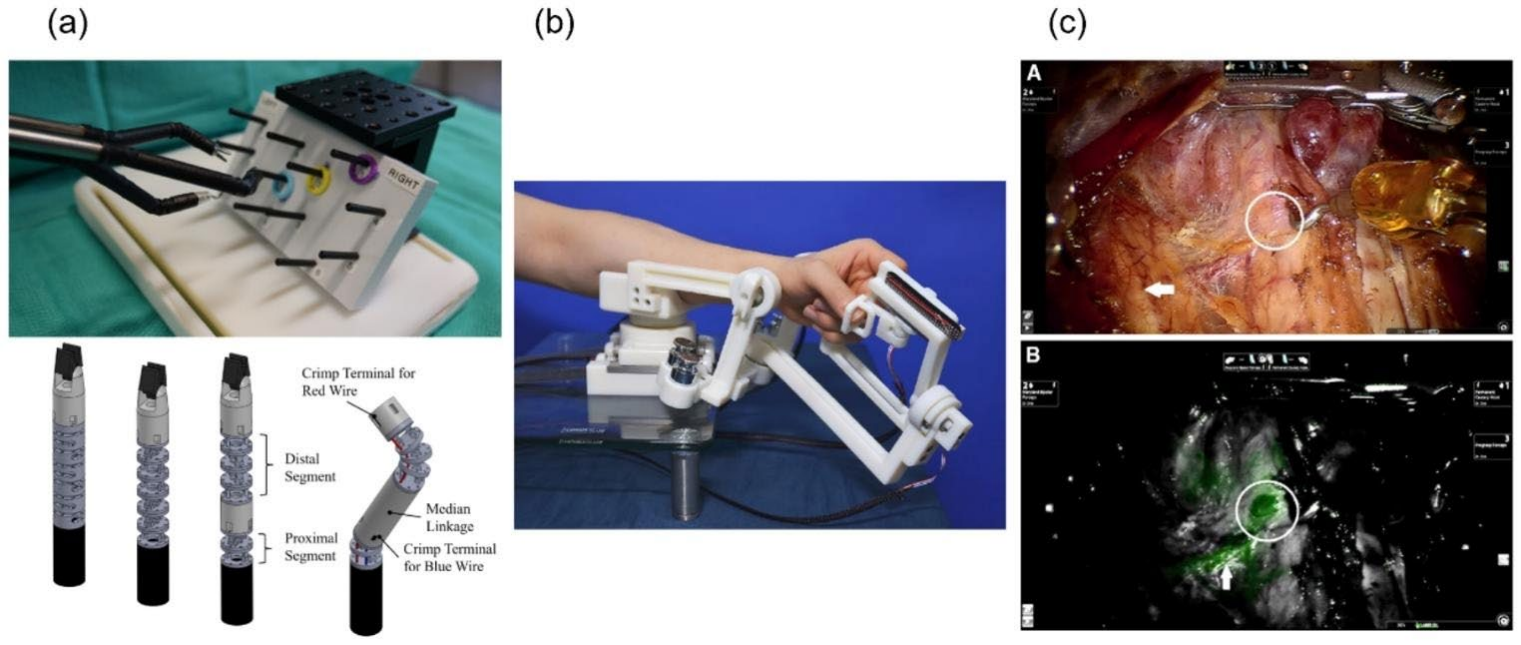
Key technologies of soft tissue surgical robots; **(a)** wire-driven surgical robotic arm [39], **(b)** master device [44], **(c)** stereoscopic views with fluorescent images [47]

Where robotic surgery directly differs from traditional laparoscopic surgery in terms of functionality is in the wire-driven articulation mechanism. The wire-driven articulation mechanisms can be miniaturized to less than 8 mm in diameter, and the wire-driving unit manipulates the surgical tool from outside the body like a puppet. Various types of mechanisms have been developed and patents have been registered, including wrist-type mechanisms and snake-like continuum mechanisms [37]. Since the joints of robotic surgical instruments are very small to solicit and feedback to sensors, modeling is essential for sensitive wire drive performance [38]. Wire driving models and real-time compensation algorithms have been studied to prevent phenomena such as slack, where the wire loses tension and backlash increases as shown in Fig. 3(a) [39].

Flexible surgical robots have a tendon-sheath mechanism (TSM) to compensate for wire length as the flexion path changes. Techniques have been studied to model and compensate for the hysteresis of the TSM [40], and more recently, artificial intelligence has been used to improve the precision of the model [41].

Improvements to the mechanism are continually being researched to enable more consistent motion and higher payloads with smaller diameters [42].

### Master device with teleoperation

The master device, mounted on the control console of the da Vinci system, allows the surgeon to maneuver the robot as intuitively as if they were sitting in a chair and holding surgical tools such as forceps. The master device can also generate reaction forces or damping to sensually convey information to the operator about the acceptable workspace and singularities of the robot arm’s posture [43]. Control algorithms can filter out the physiological tremors of the operator and implement scalable motion to enable safer and more precise surgery. This is an important factor that directly affects the operability and usability of the device as it is held and controlled by the operator. This technology is continuously being studied to realize advanced functions such as haptic feedback while having the degree-of-freedom of operation that fits the characteristics of each surgical robot as shown in Fig. 3(b) [44].

### Imaging technology

The da Vinci system acquires images with a binocular endoscope and presents them in stereoscopic images on the console’s eyepiece display, allowing surgeons to immerse themselves in the operation without the need for estimation. The stereoscopic images assisted the surgeon in performing surgical techniques and improved the quality of surgical outcomes [45]. Recent advances in electronics have led to the development of high-resolution CCD (charge coupled device) that are small enough to fit in a cell phone, but it is the optical technology, such as the lens, that ultimately determines the quality of the endoscopic view.

Furthermore, Intuitive Surgical Inc. has developed a FireFly™ feature that allows superimposed fluorescent images to be viewed on the robotic endoscope. A fluorescent substance such as indocyanine green (ICG) is injected into the body to allow visualization and identification of critical areas such as lymph nodes or the thyroid gland during endoscopic surgery [46]. There are reports of safer and improved surgical outcomes than conventional surgery using this feature as shown in Fig. 3(c) [47].

## Recent research trends and challenges

The main negative evaluation of surgical robots by medical staff is that they are uncomfortable. There is a need for robots that perform tasks precisely according to the surgeon’s intentions without interfering too much with existing techniques, surrounding equipment, or medical staff. Furthermore, recent research directions are aimed at reducing human error and improving the comfort of surgery by providing surgeons with a lot of information in real-time that cannot be given by conventional equipment. T herefore, human-robot interaction technologies such as image guidance and haptic feedback have been mainly researched recently.

Technically, the ultimate goal of surgical robotics research is autonomous surgery. Similar to autonomous vehicles, the degree of autonomy is categorized from 0 to 5 levels [48]. Currently, soft tissue surgical robots are in level 1, as they are remotely operated with the help of control technology. Research is underway to automate relatively simple procedures to reduce the burden on medical staff. Recent advances in artificial intelligence are having a major impact on autonomous surgical techniques.

As the objects and methods of surgery are vast, so are the needs of the field for new types of surgical robots. Robots for microsurgery, such as retinal surgery, are also being researched [49].

### Vision assistance with AR/VR

**Fig. 4:**
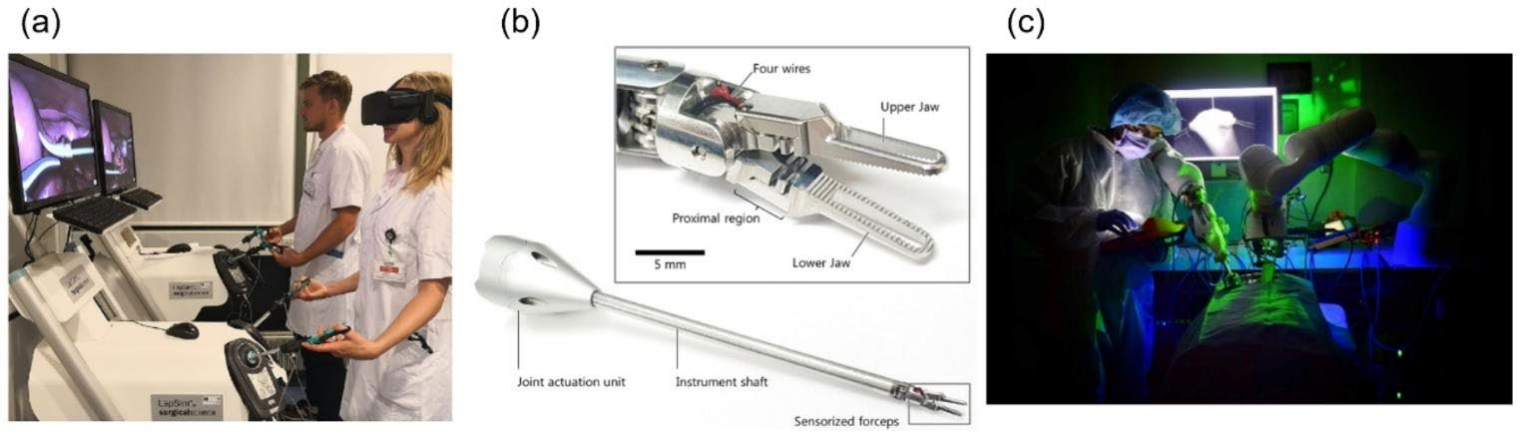
State-of-the-art technologies; **(a)** virtual reality for surgical training [51], **(b)** sensorized robotic forceps for haptic feedback [53], **(c)** autonomous laparoscopic soft tissue anastomosis [57]

One of the characteristics of laparoscopic surgery is the ease of obtaining high-resolution image information in real-time during surgery. Recently, the convergence of artificial intelligence technology and image processing technology has led to advanced research results. Surgical tools and organs are recognized and segmented within the screen, and their positions can be estimated [50]. Various information can be extracted and quantified from surgical images to evaluate surgery, and VR (Virtual Reality) can be utilized for surgical training. Image processing techniques pave the way for future advances in autonomous surgery as shown in Fig. 4(a) [51].

AR (Augmented Reality) technology, which extracts important information from images acquired by MRI (Magnetic Resonance Imaging) or CT (Computed Tomography) before surgery and merges them in real-time on the screen during surgery, is also being researched to provide the operator with information beyond fluorescent images. Image-to-image matching is a technology already in use in hard tissue surgical robots, but soft tissue surgery requires a more challenging technique that considers tissue displacement [52].

### Haptic device with force feedback

Haptic feedback technology that transmits the force exerted by the tip of a surgical robot to a master device held by the operator is an ongoing area of research. Because laparoscopic surgery uses elongated surgical tools, it relies on sensations that are insensitive compared to open surgery. Currently, surgical robots are unable to transmit the sensitive reaction forces generated by the jaws of the surgical tools to the operator. The size of the surgical tool is too small to mount the sensor on the tip, and there is not enough room for wiring. Wire-driven surgical robots are modeled similarly to long spring-damper systems and have high hysteresis, making it difficult to extrapolate small forces at the tip into wire tension.

Recently, researchers have been working to miniaturize sensors that can be attached to surgical tools to directly measure and feedback force as shown in Fig. 4(b) [53]. Other approaches include indirect force estimation and feedback based on other information that can be acquired from the surroundings, such as visual information and impedance [54].

### Autonomous surgery

Autonomous surgery is a complex and advanced technology that requires a combination of technologies such as robust mechanism design, path planning, control algorithms, and perception and localization. Current research is still preliminary. Research is underway to automatically maneuver endoscopic cameras with multi-instrument tracking algorithms during surgery to achieve optimal views [55]. Techniques are also being explored to assist the surgeon by autonomous tissue retraction to expose organs for easy access [56]. A study developed a technique to automatically perform sutures for intestinal anastomoses and compared the results of surgeons performing them manually with laparoscopic surgical tools, robotic-assisted surgery, and autonomous surgery in animal models as shown in Fig. 4(c) [57]. While there are still many issues to be resolved, researchers are taking on the challenge of developing technology for autonomous surgery.

## Conclusion

Soft tissue surgical robots have established themselves as successful professional service robots for minimally invasive surgery. It is encouraging to see a variety of products competing in a market once dominated by the da Vinci system of Intuitive Surgical Inc., and the diversification of robots, including soft tissue surgical robots and microsurgical robots. However, they have also been criticized for their high cost compared to traditional laparoscopic surgery and lack of clinical superiority or patient benefit. To overcome these challenges, research and development of surgical robots must continue, including advancing their capabilities and improving their user interface.

Communication between medical staff and engineers is crucial to the development of surgical robots. In particular, if developers don’t have a good understanding of the medical field and medical fee regulation, it’s difficult to develop a solution that will succeed in the market. Medical staff cannot provide meaningful feedback if they don’t have a good eye for engineering. Surgical robots are expected to continue to evolve in the coming years due to the increasing workload of medical staff and the demand for a higher level of care from patients.

## References

[1] Darzi SA, Munz Y (2004). The impact of minimally invasive surgical techniques. Annu Rev Med.

[2] Vierra M (1995). Minimally invasive surgery. Annu Rev Med.

[3] Cuschieri A, Dubois F, Mouiel J, Mouret P, Becker H, Buess G, Trede M, Troidl H (1991). The european experience with laparoscopic cholecystectomy. Am J Surg.

[4] Franklin ME, Rosenthal D, Abrego-Medina D, Dorman JP, Glass JL, Norem R, Diaz A (1996). Prospective comparison of open vs. laparoscopic colon surgery for carcinoma. Five-year results. Dis Colon Rectum.

[5] Zelhart M, Kaiser AM (2018). Robotic versus laparoscopic versus open colorectal surgery: towards defining criteria to the right choice. Surg Endosc.

[6] Peters BS, Armijo PR, Krause C, Choudhury SA, Oleynikov D (2018). Review of emerging surgical robotic technology. Surg Endosc.

[7] Mendes V, Bruyere F, Escoffre JM, Binet A, Lardy H, Marret H, Marchal F, Hebert T (2020). Experience implication in subjective surgical ergonomics comparison between laparoscopic and robot-assisted surgeries. J Robot Surg.

[8] Lin L, Xu C, Shi Y, Zhou C, Zhu M, Chai G, Xie L (2021). Preliminary clinical experience of robot-assisted surgery in treatment with genioplasty. Sci Rep.

[9] Cianci S, Rosati A, Rumolo V, Gueli Alletti S, Gallotta V, Turco LC, Corrado G, Vizzielli G, Fagotti A, Fanfani F, Scambia G, Uccella S (2019). Robotic single-port platform in general, urologic, and gynecologic surgeries: a systematic review of the literature and meta-analysis. World J Surg.

[10] Roh CK, Choi S, Seo WJ, Cho M, Choi YY, Son T, Hyung WJ, Kim HI (2020). Comparison of surgical outcomes between integrated robotic and conventional laparoscopic surgery for distal gastrectomy: a propensity score matching analysis. Sci Rep.

[11] Ficarra V, Novara G, Rosen RC, Artibani W, Carroll PR, Costello A, Menon M, Montorsi F, Patel VR, Stolzenburg JU, Van der Poel H, Wilson TG, Zattoni F, Mottrie A (2012). Systematic review and meta-analysis of studies reporting urinary continence recovery after robot-assisted radical prostatectomy. Eur Urol.

[12] Park JS, Choi GS, Park SY, Kim HJ, Ryuk JP (2012). Randomized clinical trial of robot-assisted versus standard laparoscopic right colectomy. Br J Surg.

[13] Gosrisirikul C, Don Chang K, Raheem AA, Rha KH (2018). New era of robotic surgical systems. Asian J Endosc Surg.

[14] Mayor N, Coppola ASJ, Challacombe B (2022). Past, present and future of surgical robotics. Trends in Urology & Men’s Health.

[15] Morton J, Hardwick RH, Tilney HS (2021). Preclinical evaluation of the Versius surgical system, a new robot-assisted surgical device for use in minimal access general and colorectal procedures. Surg Endosc.

[16] Hares L, Roberts P, Marshall K, Slack M (2019). Using end-user feedback to optimize the design of the Versius Surgical System, a new robot-assisted device for use in minimal access surgery. BMJ Surg Interv Health Technol.

[17] Haig F, Medeiros ACB, Chitty K, Slack M (2020). Usability assessment of Versius, a new robot-assisted surgical device for use in minimal access surgery. BMJ Surg Interv Health Technol.

[18] Wehrmann S, Tischendorf K, Mehlhorn T, Lorenz A, Gündel M, Rudolph H, Mirow L (2023). Clinical implementation of the Versius robotic surgical system in visceral surgery-A single centre experience and review of the first 175 patients. Surg Endosc.

[19] Raffaelli M, Gallucci P, Voloudakis N, Pennestrì F, De Cicco R, Arcuri G, De Crea C, Bellantone R (2023). The new robotic platform Hugo™ RAS for lateral transabdominal adrenalectomy: a first world report of a series of five cases. Updates Surg.

[20] Gueli AS, Chiantera V, Arcuri G, Gioè A, Oliva R, Monterossi G, Fanfani F, Fagotti A, Scambia G (2022). Introducing the New Surgical Robot HUGO™ RAS: system description and docking settings for gynecological surgery. Front Oncol.

[21] Totaro A, Campetella M, Bientinesi R, Gandi C, Palermo G, Russo A, Aceto P, Bassi P, Sacco E (2022). The new surgical robotic platform HUGO™ RAS: System description and docking settings for robot-assisted radical prostatectomy. Urologia.

[22] Kastelan Z, Hudolin T, Kulis T, Knezevic N, Penezic L, Maric M, Zekulic T (2021). Upper urinary tract surgery and radical prostatectomy with Senhance® robotic system: single center experience-first 100 cases. Int J Med Robot.

[23] Chang KD, Abdel Raheem A, Choi YD, Chung BH, Rha KH (2018). Retzius-sparing robot-assisted radical prostatectomy using the Revo-i robotic surgical system: surgical technique and results of the first human trial. BJU Int.

[24] Liatsikos E, Tsaturyan A, Kyriazis I, Kallidonis P, Manolopoulos D, Magoutas A (2022). Market potentials of robotic systems in medical science: analysis of the Avatera robotic system. World J Urol.

[25] Hinata N, Yamaguchi R, Kusuhara Y (2022). Hinotori surgical robot system, a novel robot-assisted surgical platform: preclinical and clinical evaluation. Int J Urol.

[26] MicroPort. MicroPort® Toumai® Surgical Robot Receives NMPA Approval, Becoming the First Commercialized Four-Arm Laparoscopic Surgical Robot Developed in China. https://microport.com/news/microport-toumai-surgical-robot-receives-nmpa-approval-becoming-the-first-commercialized-four-arm-laparoscopic-surgical-robot-developed-in-china. Accessed 27 Jan 2022.

[27] Morelli L (2016). Da Vinci single site© surgical platform in clinical practice: a systematic review. Int J Med Robot Comp Assist Surg.

[28] Lee SR, Roh A, Jeong K, Kim SH, Chae HC, Moon HS (2021). First report comparing the two types of single-incision robotic sacrocolpopexy: single site using the da Vinci Xi or Si system and single port using the da Vinci SP system. Taiwan J Obstet Gynecol.

[29] Kim JM, Lee SM, Seol A, Song JY, Ryu KJ, Lee S, Park HT, Cho HW, Min KJ, Hong JH (2023). Comparison of surgical outcomes between single-port laparoscopic surgery and da vinci single-port robotic surgery. J Pers Med.

[30] Van Abel KM, Yin LX, Price DL, Janus JR, Kasperbauer JL, Moore EJ (2020). One-year outcomes for da vinci single port robot for transoral robotic surgery. Head Neck.

[31] Kneist W, Stein H, Rheinwald M (2020). Da Vinci Single-Port robot-assisted transanal mesorectal excision: a promising preclinical experience. Surg Endosc.

[32] Seeliger B, Swanström LL (2020). Robotics in flexible endoscopy: current status and future prospects. Curr Opin Gastroenterol.

[33] Lang S, Mattheis S, Hasskamp P (2017). A european multicenter study evaluating the flex robotic system in transoral robotic surgery. Laryngoscope.

[34] Reisenauer J, Simoff MJ, Pritchett MA (2022). Ion: technology and techniques for shape-sensing robotic-assisted bronchoscopy. Ann Thorac Surg.

[35] Sean W. FDA clears J&J’s Ethicon’s Monarch surgical robot for urology procedures. Mass Device. https://www.massdevice.com/fda-clears-jjs-ethicons-monarch-surgical-robot-for-urology-procedures/utm_source=TrendMD&utm_medium=cpc&utm_campaign=Mass_Device_TrendMD_0. Accessed 2 May 2022.

[36] Agrawal A, Hogarth DK, Murgu S (2020). Robotic bronchoscopy for pulmonary lesions: a review of existing technologies and clinical data. J Thorac Dis.

[37] Jelínek F, Arkenbout EA, Henselmans PWJ, Pessers R, Breedveld P (2015). Classification of joints used in steerable instruments for minimally invasive surgery—a review of the state of the art. ASME J Med Devices.

[38] Webster R, Jones B (2010). Design and kinematic modeling of constant curvature continuum robots: a review. Int J Robot Res.

[39] Jin S, Lee SK, Lee J, Han S (2019). Kinematic model and real-time path generator for a wire-driven surgical robot arm with articulated joint structure. Appl Sci.

[40] Do TN, Tjahjowidodo T, Lau MWS, Yamamoto T, Phee SJ (2014). Hysteresis modeling and position control of tendon-sheath mechanism in flexible endoscopic systems. Mechatronics.

[41] Kim D, Kim H, Jin S (2022). Recurrent neural network with preisach model for configuration-specific hysteresis modeling of tendon-sheath mechanism. IEEE Robot Autom Lett.

[42] Kim J, Kwon S, Moon Y, Kim K (2022). Cable-movable rolling joint to expand workspace under high external load in a hyper-redundant manipulator. IEEE/ASME Trans Mechatron.

[43] Preusche C, Ortmaier T, Hirzinger G (2002). Teleoperation concepts in minimal invasive surgery. Control Eng Pract.

[44] Shim S, Kang T, Ji D (2016). An all-joint-control master device for single-port laparoscopic surgery robots. Int J CARS.

[45] Willis DL, Gonzalgo ML, Brotzman M, Feng Z, Trock B, Su LM (2012). Comparison of outcomes between pure laparoscopic vs robot-assisted laparoscopic radical prostatectomy: a study of comparative effectiveness based upon validated quality of life outcomes. BJU Int.

[46] Meershoek P, KleinJan GH, van Willigen DM (2021). Multi-wavelength fluorescence imaging with a da Vinci Firefly—a technical look behind the scenes. J Robot Surg.

[47] Yu HW, Chung JW, Yi JW (2017). Intraoperative localization of the parathyroid glands with indocyanine green and Firefly (R) technology during BABA robotic thyroidectomy. Surg Endosc.

[48] Yang GZ (2017). Medical robotics—Regulatory, ethical, and legal considerations for increasing levels of autonomy. Sci Robot.

[49] Ebrahimi A, Sefati S, Gehlbach P (2022). Simultaneous online registration-independent stiffness identification and tip localization of surgical instruments in robot-assisted eye surgery. IEEE Trans on Robotics.

[50] Zinchenko K, Song KT (2021). Autonomous endoscope robot positioning using instrument segmentation with virtual reality visualization. IEEE Access.

[51] Frederiksen JG, Sørensen SMD, Konge L (2020). Cognitive load and performance in immersive virtual reality versus conventional virtual reality simulation training of laparoscopic surgery: a randomized trial. Surg Endosc.

[52] Golse N, Petit A, Lewin M (2021). Augmented reality during open liver surgery using a markerless non-rigid registration system. J Gastrointest Surg.

[53] Kim U, Kim YB, So J, Seok DY, Choi HR (2018). Sensorized surgical forceps for robotic-assisted minimally invasive surgery. IEEE Trans Ind Electron.

[54] Chua Z, Okamura AM. Characterization of real-time haptic feedback from multimodal neural network-based force estimates during teleoperation., Systems J. 2022;pp. 1471–1478. 10.1109/IROS47612.2022.9981662.

[55] Li L, Li X, Ouyang B (2021). Autonomous multiple instruments tracking for robot-assisted laparoscopic surgery with visual tracking space vector method. IEEE ASME Trans Mechatron.

[56] Attanasio A (2020). Autonomous tissue retraction in robotic assisted minimally invasive surgery – a feasibility study. IEEE Robot Autom Lett.

[57] Saeidi H, Opfermann JD, Kam M, Wei S, Leonard S, Hsieh MH, Kang JU, Krieger A. Autonomous robotic laparoscopic surgery for intestinal anastomosis. Sci Rob. 2022;7(62). 10.1126/scirobotics.abj2908. 10.1126/scirobotics.abj2908 PMC8992572 35080901

[58] Hutchins AR, Manson RJ, Lerebours R (2019). Objective assessment of the early stages of the learning curve for the senhance surgical robotic system. J Surg Educ.

[59] Medicaroid.Regulatory. Approval Application Filed with the Health Sciences Authority in Singapore for the “hinotoriTM Surgical Robot System”. https://www.medicaroid.com/en/release/pdf/230508_en.pdf. Accessed 8 May,2023.

[60] Koukourikis P, Rha KH (2021). Robotic surgical systems in urology: what is currently available?. Invest Clin Urol.

